# Melanotic neuroectodermal tumor of infancy arising in the skull: a case report

**DOI:** 10.11604/pamj.2024.47.62.42413

**Published:** 2024-02-12

**Authors:** Hajar Hamadi, Taib El Amrani El Idrissi, Ayman Gallouli, Lamia Benantar, Khalid Aniba

**Affiliations:** 1 Department of Neurosurgery, Ibn Tofail Hospital, Mohammed VI^th^ University Hospital, Faculty of Medicine and Pharmacy in Marrakech, Cadi Ayyad University, Marrakech, Morocco

**Keywords:** Melanotic neuroectodermal tumor, calvaria, skull, melanotic progonoma, case report

## Abstract

Melanotic neuroectodermal tumor of infancy is a rare and usually benign neoplasm occurring in children of young age. This pigmented tumor typically presents in the head and neck region, but other locations may be involved. We report in this article a rare case of a 3-month-old girl presenting with a slowly growing mass localized in the anterior fontanelle. The patient's magnetic resonance imaging (MRI) showed a mass extending both extracranial and intracranial, and compressing the adjacent structures. The patient underwent subtotal resection of the mass and a histological study confirmed the diagnosis of melanotic neuroectodermal tumor of infancy. The patient presented later on with a recurrence. An early diagnosis and surgical management for these tumors remain the only guarantees to limit the progression and prevent their recurrence and metastasis.

## Introduction

Melanotic neuroectodermal tumor of infancy (MNTI), is a rare and usually benign neoplasm occurring in children of young age [1]. Described initially by Krompecher in 1918, it was referred to as “Congenital melanocarcinoma” as it was believed to be derived from pigmented odontogenic or epithelial cells. Due to the difficulties in deciding its cellular origin, it was called in the old nomenclature by many terms such as melanotic epithelial odontoma, pigmented teratoma, atypical melanoblastoma, melanotic adamantinoma, pigmented epulis, retinal anlage tumor, and melanotic progonoma. The discovery of elevated urinary excretions of vanillylmandelic acid (VMA) by Borello and Gorlin in 1966 was crucial for the understanding of the histopathology of the tumor which was suspected to be of neural crest origin, leading to the first use of the term MNTI [1-3]. This pigmented tumor typically presents in the head and neck region with 70% of cases occurring in the maxilla, 11% in the skull and 6% in the mandible, other locations maybe involved such as the brain, meninges, epididymis, mediastinum, ovary, uterus, and bones of the extremities [2,4,5]. In the skull; melanotic neuroectodermal tumor of infancy usually arises around sutures and about 50% of the cases occur around the parieto-occipital region and around the anterior fontanelle [2-5]. Although most cases occur in infancy with a male predilection, some rare cases have been reported in older children and adults [3]. Despite its benign histological characteristics, this neoplasm may present a locally aggressive behavior, with a rapid progression and destructive invasion of adjacent structures causing deformities, as well as a high recurrence rate with a possibility of malignant transformation, and metastasis [3,5]. We report here a case of recurrent benign MNTI of the skull, with a review and analysis of literature on MNTI with cranial involvement.

## Patient and observation

**Patient information:** the patient is a 3-month-old girl born to nonconsanguineous parents. The history of pregnancy and birth was uneventful, and she had no significant family or personal medical history. She was referred to the Neurosurgery department at Ibn Tofail Hospital - Mohamed VI^th^ University Hospital of Marrakech with an 8-week history of rapidly growing midline scalp.

**Figure 1:**
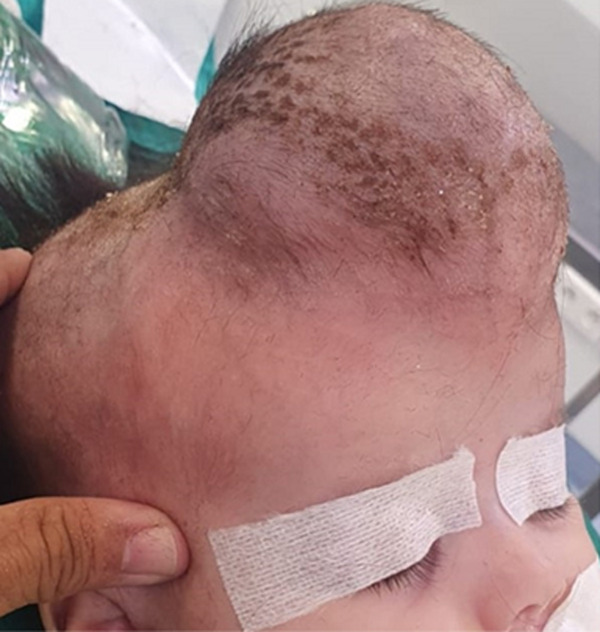
preoperative image of the frontal mass

**Clinical findings**: local examination of the mass showed a firm nonmobile mass with no skin changes, seemingly adherent to the adjacent structures, located at the midline frontal region and measuring 5.7 in diameter (Figure 1). Neuropsychomotor development (NPMD) was normal for the infant´s age, with no evidence of neurological deficit.

**Timeline of current episode:** the mass first appeared approximately 3 weeks after birth. The parents did not seek medical help, as the mass was small and had no significant consequences of the infant´s development. The tumefaction progressively increased in size over a period of 8 weeks, reaching a significant size, which lead the parents to seek medical attention with a primary care physician who referred them to our department.

**Figure 2:**
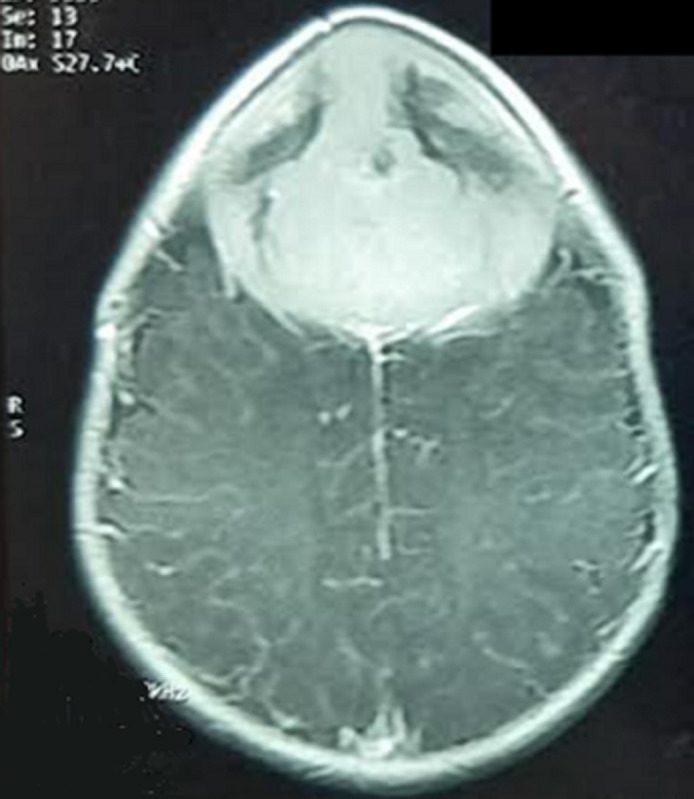
cerebral magnetic resonance imaging axial plane in T1-weighted sequence with contrast

**Figure 3:**
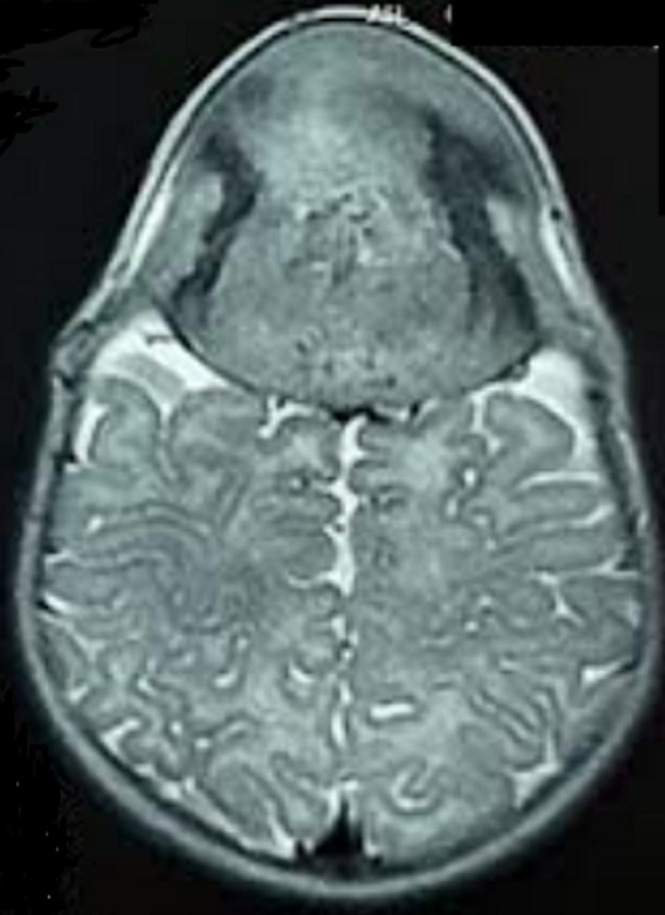
cerebral magnetic resonance imaging axial plane in T2-weighted sequence

**Figure 4:**
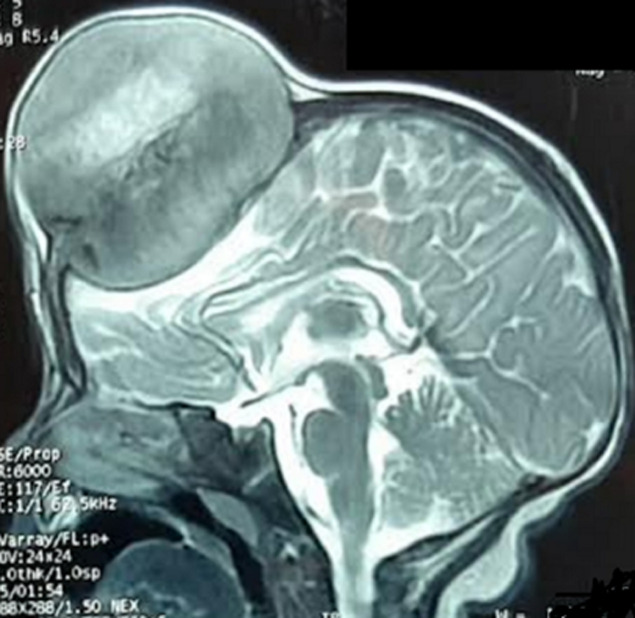
cerebral magnetic resonance imaging sagittal plane in T2-weighted sequence

**Diagnostic assessment:** magnetic resonance imaging of the brain revealed a well-defined extra-axial lesion centered at the anterior fontanelle measuring 64x61x71 millimeters in isosignal T1 and moderate hypersignal in T2, extending both extracranial and intracranial, and compressing the adjacent frontal brain parenchyma from which it was separated with a thin layer of cerebrospinal fluid. The lesion showed an intense enhancement after gadolinium injection, with central necrosis. Moreover, it was responsible for bone lysis and thickening of the contiguous meninges (Figure 2, Figure 3, Figure 4).

**Diagnosis:** diagnostic hypotheses at this stage included sarcoma, granuloma or meningioma.

**Figure 5:**
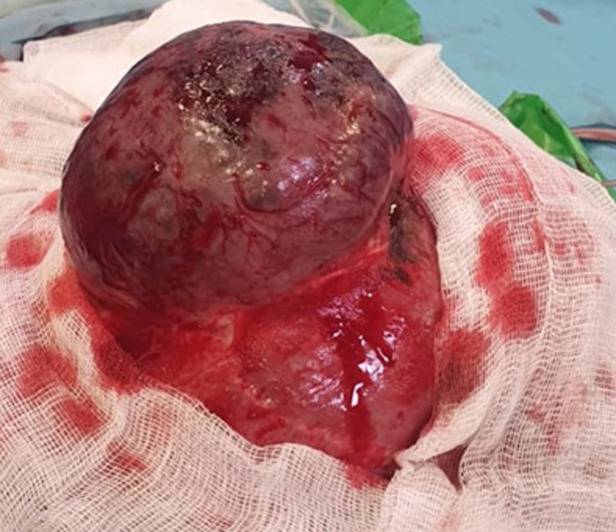
intraoperative image of the mass

**Therapeutic interventions:** prognosis and treatment plan were advised to the parents, and the patient underwent subtotal surgical removal of the lesion. After general anesthesia, a coronal incision was made to allow for better access and exposure of the lesion. Intraoperatively, the lesion was firm and blackish (Figure 5). It was adherent to the dura matter and to the superior sagittal sinus, which did not allow for a complete resection of the tumor. The lesion was minimally vascularized, invading the dura matter and coming in close contact with the parenchyma without any signs of invasion on exploration. The intervention went smoothly, and no complications were observed during the postoperative period.

**Figure 6:**
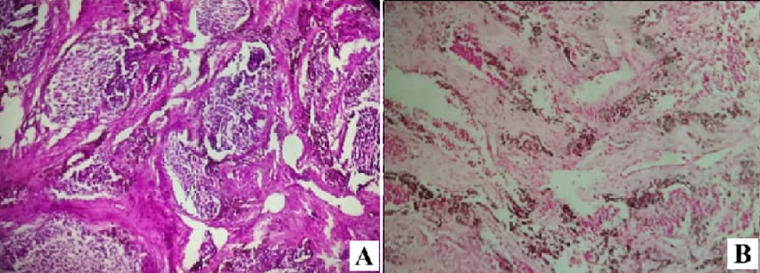
hematoxylin-eosin staining x200; A) and immunochemical staining; B) of the resected specimen

**Figure 7:**
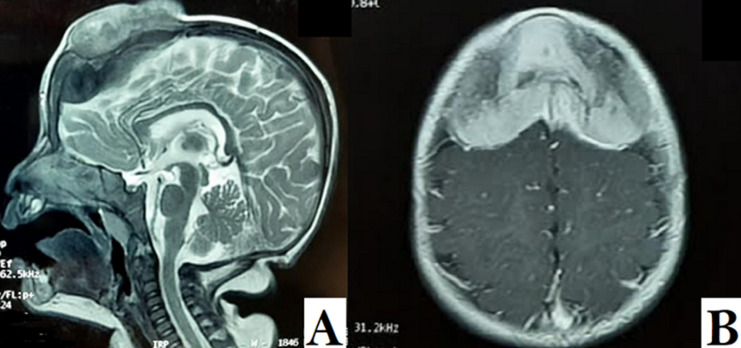
control cerebral magnetic resonance imaging of the patient at 6 months follow-up

**Follow-up and outcome of interventions:** gross pathology examination of the resected specimen revealed a partially calcified rubbery gray-white mass. Cross-section microscopy showed a biphasic tumor proliferation made of large epithelial cells with hyperchromic rounded nuclei, melanotic deposits and eosinophile cytoplasm arranged in lobules and sheets, and small tumor cells with hyperchromatic nuclei and scant cytoplasm. The cells were surrounded by a dense fibrotic stroma. This tumoral proliferation invaded the adjacent bone tissue, with no signs of necrosis or vascular emboli. Immunohistochemically, the large epithelioid cells were intensely positive for the melanocytic marker, pancytokeratin and HMB-45, and negative -or presence of traces-for synaptophysin, chromogranin A and glial fibrillary acidic protein. Ki-67 proliferation index was at 30%. The previously mentioned characteristics on the pathology examination confirmed the diagnosis of MNTI (Figure 6). On her 6 months follow-up, the patient showed a bulging firm mass at the frontal fontanel approximately 2.6cm in diameter consistent with a recurrence which was confirmed by a control MRI of the brain (Figure 7). In light of the patient´s evolution and normal neurologic examination, we decided that a conservative treatment approach would be best. The patient was referred to the oncology department, which decided that no further chemo or radiotherapy was needed. She was seen on 8 months, 10 months and 12 months follow-up, and the examination showed a stable mass with no increase in volume as well as normal NPMD.

**Patient perspective:** the parents were satisfied with the results of the surgical resection. The management of the recurrence was discussed with the parents, and the risks and advantages of a second surgical intervention were thoroughly explained. A conservative treatment with further follow-up with the oncology team was deemed the right approach.

**Informed consent:** prognosis and diagnosis were explained to the parents and informed consent was obtained.

## Discussion

Melanotic neuroectodermal tumor of infancy (MNTI) is a very rare neoplasm with only 93 reports of MNTI of the skull in the last century, making this location even more unusual [1,3,5]. A male predilection with a median age at presentation of 4.5 months was observed in MNTI arising in the skull which reflects the whole population of MNTI [1,3,5,6]. The presentation of MNTI in the skull is that of a rapidly growing, firm, fixed, painless mass, typically occurring in the anterior fontanel region in newborns and infants under the first year of life [1,5,7]. It usually occurs as one lesion but reports of multicentricity have been reported, yet none of these reports included MNTI of the skull [5,8]. Computed tomography scan shows a well-defined hyperdense mass with adjacent bone destruction and thickening due to reactive sclerosis, while the MRI findings vary from hypointense to hyperintense and a mixed signal on T1-weighted imaging, and an isointense/hypointense signal on T2-weighted imaging. However, post contrast with gadolinium images showed strong and homogeneous enhancement [2,5]. This is a challenging issue to a neurosurgeon, distinguishing MNTI of the skull from other malignant tumors is a crucial step to management especially in the pediatric population where the differential diagnosis includes Ewing sarcoma, neuroblastoma, rhabdomyosarcoma and lymphoma. Due to the variability in imaging findings, tissue biopsy with a histopathological examination is necessary to confirm the diagnosis. An increased level of vanillylmandelic acid (VMA) was suggested to help in the differential diagnosis but low incidence of increased VMA in MNTI of the skull may not be useful to narrowing and confirming the diagnosis [2,5,7].

The histologic hallmark of MNTI was the biphasic population of cells composed of larger melanogenic cells and smaller neuroblast like cells [1,5,8]. Immunohistochemical analysis can be used to establish the diagnosis [5]. Melanotic neuroectodermal tumors of infancy are generally histologically benign, but a malignancy rate of 6.5% has been reported mainly in other locations (Brain, maxilla) [5,8]. In our review, no case of malignant transformation or metastasis was reported. The standard treatment for these tumors is complete surgical resection with tumor-free margins [1,5]. Chemotherapy and radiotherapy have been used in initially inoperable tumors or in the case of recurrences [1,9]. Some articles describe the use of chemotherapy after incomplete or partial resections, but this particular indication is cause for controversy with conflicting reports on its efficacy of the disease evolution [2,5,9]. In our review, 83% of the patient underwent total surgical removal of the tumor, with only 8 cases requiring post-operative chemotherapy and radiotherapy for a recurrent MNTI. Only 3 cases underwent a course of chemotherapy prior to surgical removal for cases of inoperable sizeable tumors. The recurrence rate of these tumors tends to be high, and is believed to be related to incomplete excision of the primary tumor, seeding during surgery, or tumor multicentricity. It has been reported to vary between 15% and 45% [1,2,5,9]. Ren *et al*. review relates the high recurrence rate to certain risk factors including an age at diagnosis of older than 1 year, the greatest dimension of the tumor being >5cm, subtotal surgical resection or nonsurgical treatment, metastasis, and malignant transformation [5].

## Conclusion

Melanotic neuroectodermal tumors of infancy of the skull are rare histologically benign tumors of neuroectodermal origin with rapid locally aggressive behavior. We presented a case of a 3-month-old with a recurrent MNTI arising in the anterior fontanelle treated surgically. An early diagnosis and surgical management remain the only guarantees to limit the tumor´s progression and prevent its recurrence and metastasis. But, despite the clear course of action regarding the management of this disease, the rarity of its occurrence poses a diagnostic challenge to neurosurgeons which could delay the treatment resulting in a less favorable outcome.
